# *Stenotrophomonas tuberculopleuritidis* sp. nov., a novel pathogenic *Stenotrophomonas* species isolated from tuberculous pleurisy patient

**DOI:** 10.3389/fcimb.2025.1629703

**Published:** 2025-08-07

**Authors:** Zelin Yu, Boqing Xu, Ruibai Wang

**Affiliations:** National Key Laboratory of Intelligent Tracking and Forecasting for Infectious Disease, National Institute for Communicable Disease Control and Prevention, Chinese Centre for Disease Control and Prevention, Beijing, China

**Keywords:** stenotrophomonas, tuberculous pleurisy, multiple-drug-resistant, sputum, patient, pathogen

## Abstract

**Introduction:**

*Stenotrophomonas* represents a group of bacteria that exhibit significant value in industrial and agricultural applications, while also posing pathogenic risks to humans. 704A1^T^ was isolated from a patient with tuberculous pleurisy. Its 16S rRNA sequence showed the highest homology (99.72%) with a *Stenotrophomonas* strain without defined species classification. It is necessary to clarify the species 704A1^T^ belonging to and its potential pathogenicity to humans.

**Methods:**

Systematical evaluations including phenotypic and biochemical characteristics, antibiotic susceptibility, genomic sequencing were conducted. The pathogenicity and immunological characteristics were tested by intranasally inoculated C57BL/6J mice.

**Results:**

704A1^T^ is Gram negative rod-shaped bacterium with flagella at single extreme. Showing highly similar with *S. maltophilia*, 704A1^T^ also displayed distinct characteristic peaks in fatty acid profiling and MALDI-TOF analysis. 704A1^T^ was resistance to 21 antibiotics, including four anti-tuberculosis drugs: rifampicin, streptomycin, rifabutin, and cycloserine. The average nucleotide identity (ANI) values of 704A1^T^ compared to defined *Stenotrophomonas* species ranged from 80.03% to 89.6%, below than both the commonly accepted 95%-96% ANI threshold for prokaryote species and the 95% threshold suggested for *Stenotrophomonas*. Though no mortality was observed, 704A1^T^ could cause severe consolidation in murine lung tissue and has the ability of hematogenous dissemination.

**Conclusion:**

Results supported the classification of 704A1^T^ (=GDMCC 1.4133^T^) as a novel species within the genus *Stenotrophomonas*, for which the name *Stenotrophomonas tuberculopleuritidis* sp. nov. is proposed. 704A1^T^ is a multi-antibiotic resistance strain with potentially stronger pathogenicity than *S. maltophilia* and requires more clinical attention. The isolation of 704A1^T^ underscored the importance of sustained surveillance and taxonomic clarity of *Stenotrophomonas* species emerging from clinical environments.

## 1 Introduction

*Stenotrophomonas* exhibits a wide range of habitats, including natural environment, patients, animals and plants. The common characteristics of *Stenotrophomonas* species are as follows: 1.Strong biofilm formation ability, enabling the formation of biofilms on various abiotic surfaces and tissues; 2. Extensive intrinsic and acquired multidrug resistance (mediated by efflux pumps, β-lactamases, and horizontal gene transfer), which confers resistance to clinically commonly used drugs such as cephalosporins and makes it as an emerging superbug (Sanchez, 2015; Brooke et al., 2017; Gil-Gil et al., 2020; Kumar et al., 2020); 3. High genotypic and phenotypic variability, even within the same species, which is the reason for the several changes in the taxonomic classification of the genus *Stenotrophomonas* and its species (Palleroni and Bradbury, 1993); 4. Possessing bacterial structures such as lipopolysaccharides, flagella, and pili, as well as type IV secretion systems; 5. Abundant extracellular enzymes, including proteases, esterases, phospholipases, extracellular nucleases, hyaluronidases, heparinases, hemolysins, and siderophores, etc (Trifonova and Strateva, 2019); 6. Capability to produce various active bactericidal factors, including proteolytic enzymes acting on *Ralstonia solanacearum* (Elhalag et al., 2016), antifungal substances Maltophilin (Jakobi et al., 1996) and Xanthobaccin (Nakayama et al., 1999), serine proteases against nematodes (Huang et al., 2009), broad-spectrum antibacterial R-type phage tail-like bacteriocins Maltocin P28 (Liu et al., 2013), S16 (Chen et al., 2019), and modular bacteriocins Stenocins (Paškevičius et al., 2022).

Therefore, *Stenotrophomonas* is a fascinating bacterium with a dual functional role. On the one hand, *Stenotrophomonas* has robust resistance to biotic and abiotic stresses, including plant pathogenic fungi and bacteria (Kumar et al., 2023). These bacteria engage in complex interactions with other microorganisms on plant surfaces and within soil through biofilm formation. They inhibit plant fungal pathogens and viruses while modulating rhizosphere microbial communities via mechanisms such as extracellular enzyme-mediated decomposition, iron nutrient competition, and more (Kumar et al., 2023). Against crop pests like *Spodoptera litura*, they trigger the accumulation of plant hormones (e.g., jasmonic acid) and upregulate jasmonic acid-responsive gene transcription (Ling et al., 2022). For plant growth promotion, they produce auxins and hydrogen cyanide, solubilize phosphorus/potassium salts, fix atmospheric nitrogen, and degrade compounds like geosmin, explosive pollutants (hexahydro-1, 3, 5-trinitro-1, 3, 5–triazine, RDX), keratin, macrocyclic hydrocarbons, and nitrophenols (Gao et al., 2013). These capabilities enable *Stenotrophomonas* to remediate agricultural soils by eliminating chemical pesticides, insecticides, and diverse environmental pollutants. Therefore, most *Stenotrophomonas* species are considered to have significant agronomic and industrial application values, established as plant growth-promoting rhizobacteria (PGPR), emerging biocatalysts for environmental biodegradation, and sustainable substitutes for synthetic fungicides.

While on the other hand, these common characteristics of *Stenotrophomonas* function as virulence factors in host colonization and infection. For instance, they degrade the extracellular matrix of tissue cells in the host respiratory system, connective tissues (especially collagen and fibronectin), neutrophil extracellular traps, serum immune proteins, etc., endowing *Stenotrophomonas* with high invasiveness to inactivate/evade the host immune defense system. They damage pulmonary epithelium, causing fulminant hemorrhagic pneumonia; induce intense inflammatory immune responses; mediate host tissue adhesion and form biofilms on abiotic surfaces like medical devices, enhancing the transmission of nosocomial infections; and synergistically exacerbate infections by other pathogens. Therefore, *Stenotrophomonas*, typified by *S. maltophilia*, is an emerging global opportunistic pathogen with growing clinical threat (Brooke, 2012). They can cause severe infections in multiple organs and tissues, including bloodstream and pneumonia. Notably, its bacteremia is associated with a mortality rate as high as 65% (Tan et al., 2014). *S. maltophilia* infection has be seen as a life-threatening disease in ICU patients, cancer patients, and immunocompromised individuals. Alongside *Achromobacter xylosoxidans*, it serves as a marker for the progression to severe lung diseases, holding comparable pathophysiological significance to *Pseudomonas aeruginosa*—the hallmark pathogen in cystic fibrosis patients (Menetrey et al., 2021). Consequently, the World Health Organization has classified *S. maltophilia* among the world’s most concerning emerging multidrug-resistant (MDR) bacteria. Following extended-spectrum β-lactamase (ESBL)-producing *Enterobacteriaceae*, carbapenem-resistant *Enterobacteriaceae*, and refractory *Pseudomonas aeruginosa*, treatment guidelines from the Infectious Diseases Society of America (IDSA) list *S. maltophilia* alongside AmpC β-lactamase-producing *Enterobacteriaceae* and carbapenem-resistant Acinetobacter baumannii as the three pathogens causing severe hospital-acquired infections with significant morbidity and mortality across U.S. healthcare settings (Tamma et al., 2022).

Among the definite S*tenotrophomonas* species, only seven species are considered to be human pathogens or have a history of isolation from human samples or clinical environments, namely *S.maltophilia*, *S. sepilia* (Gautam et al., 2021), *S. pavanii* (Iebba et al., 2018), *S. acidaminiphila* (Jezek et al., 2020; Zhang et al., 2022), *S. rhizophila* (Chao et al., 2022), *S. riyadhensis* (Macori et al., 2024) and *S*. *pigmentate* (Li et al., 2024). Notably, in our prior study on the co-infection/co-isolation of *Stenotrophomonas* and *Mycobacterium* (Li et al., 2023), nine *Stenotrophomonas* isolates identified via 16S rRNA sequencing were assigned to three species: *S. maltophilia*, *S. rhizophila*, and *S. pigmentate* (Li et al., 2024). However, one strain, 704A1T, remained unassigned to a specific species. The 16S rRNA sequence of 704A1^T^ exhibited the highest homology (99.72%) with *Stenotrophomonas* sp. strain DoB6 (JQ359085.1), and its *recA* gene showed the highest similarity (99.72%) to *Stenotrophomonas* sp. strain ZAC14D2_MKIMI4 (KX896038.1). Both strains have not a defined species annotation. Because this strain was also isolated from tuberculosis (TB) patient, indicating its potential pathogenicity and clinical importance to humans, it was evaluated systematically in this study to clarify its classification in the genus *Stenotrophomonas* and the main phenotypic, genetic and pathogenic characteristics.

## 2 Materials and methods

### 2.1 Strain isolation and identification

Sputum sample was collected from the TB clinic of Chaoyang District Center for Disease Control and Prevention (39°89’ N, 116°40’ E) with traditional N-acetyl-1-cysteine-4% NaOH-phosphate alkaline decontamination treatment. The colonies on the 7H10 + 10% oleic acid-albumin-dextrose-catalase (OADC) agar plate were identified as *Stenotrophomonas* strains by amplification and sequencing with16S rRNA universal bacterial primers (16S-U: 5’AGA GTT TGA TCM TGG CTC AG 3’and 16S-L: 5’ CCG TCA ATT CMT TTR AGT TT 3’) (Li et al., 2023).

### 2.2 Phenotypic and biochemical characteristics

Besides 7H10 + 10% OADC agar, the growth abilities of this strain on four kinds of medium, tryptone soy (TSA; Difco), Luria-Bertani (LB; Difco), columbia blood agar (OXOID) and Mueller-Hinton (MH; Difco), and under conditions of 15-37°C, pH range 5-10, and 0-5% NaCl concentration were tested. Cell modality was observed with optical microscope and transmission electron microscope (HT7700, Hitachi, Japan). Utilization of carbon sources and enzyme production were tested with the API 20NE, API ZYM and API 50 CH test kits (20050, 50300, 25200, BioMerier France, France) at 28°C and the endpoints were 48 h, 4 h and 48 h respectively according to the manufacturer’s manual.

### 2.3 Fatty acids analysis and matrix-assisted laser desorption/ionization time-of-flight mass spectrometry

Cellular fatty acids were analysed using the Sherlock Microbial Identification System (MIDI) with the MIDI Sherlock software program (version 6.3) and RTSBA6 (6.21) library. MALDI-TOF was conducted on Autoflex speed TOF/TOF (Bruker Daltonics GmbH, Germany) and automatically interpreted by MALDI Biotype IVD2.3 (5989) analysis software. Identification score above 2.0 is cutoff value of intra species, and a score of 1.7-2.0 is intra genus.

### 2.4 Antibiotic susceptibility testing

The BD Phoenix™ NMIC 413 Panels on BD Phonenix-100 equipment (BD, Sparks, MD, USA), the customised AST plate for Chinese Pathogen Identification Net (CHNENF, Trek Diagnostic Systems Ltd, West Sussex, United Kingdom) and the Sensititre^™^ MYCOTB MIC plate (Trek Diagnostic Systems, Cleveland, OH, USA) were used to determine the strain’s sensitivity to drugs commonly used for Gram-negative strains and 12 anti-TB drugs. The minimum inhibitory concentrations (MIC) were judged according to the standards of the Clinical and Laboratory Standard Institute (CLSI) (CLSI, 2018; CLSI, 2023) and manufacturer’s instructions.

### 2.5 Whole genome sequencing and gene function analysis

Genome of 704A1^T^ was sequenced using a Pacbio sequel II and DNBSEQ platform under four SMRT cells Zero-Mode. Self-correction, single-base correction and gene prediction were performed sequentially by the program Canu, GATK and Glimmer3 with Hidden Markov model. Genome components were predicted using localized software, tRNAscan SE (version:1.3.1), Tandem Repeats Finder (version:4.04), PhiSpy (version: 4.2.21), etc. Gene function scanning was performed based on the databases listed below, KEGG (Kyoto Encyclopedia of Genes and Genomes, http://www.kegg.jp), COG (Clusters of Orthologous Groups, http://www.ncbi.nlm.nih.gov), NR (Non-Redundant Protein Database databases, ftp://ftp.ncbi.nih.gov/blast/db/FASTA/nr.gz), Swiss-Prot (http://ngdc.cncb.ac.cn), GO (Gene Ontology, http://www.geneontology.org), TrEMBL, EggNOG (http://eggnog.embl.de), VFDB (Virulence Factors of Pathogenic Bacteria, http://www.mgc.ac.cn), ARDB (Antibiotic Resistance Genes Database, http://www.argodb.org/), CAZy (Carbohydrate-Active enZYmes Database, http://www.cazy.org) and T3SS (Type III secretion system effector protein, http://effectivedb.org).

### 2.6 Pairwise average nucleotide identity comparison and phylogenetic analysis

16S rRNA gene sequence, ANI and core gene profiles of 704A1^T^ were comprehensively compared with those of all 33 validated *Stenotrophomonas* species included in the Taxonomy up to 2024. 16S rRNA gene sequences of the type strains of each species were obtained from LPSN (https://lpsn.dsmz.de/) by their GenBank accessions, except for *Stenotrophomonas muris*, for which the 16S rRNA gene sequence was retrieved from its reference genome GCF_024621935.1 (gene locus NSA55_RS22000). Multiple sequence alignment was performed using Muscle v5.3 (https://drive5.com/muscle/) (Edgar, 2022). Reference genomes for each species were obtained from the NCBI database (except for *S. panacihumi*). The reference genome of *Xanthomonas campestris* (GCF_013388375.1) was used as an outgroup. Pairwise ANI calculations were performed using FastANI v1.34 (Jain et al., 2018) (https://github.com/ParBLiSS/FastANI), followed by average linkage hierarchical clustering through Python scripts, with results saved in Newick format. The valid value output threshold of FastANI is >78%. Single-copy orthologous protein sequences were identified using OrthoFinder v2.5.5 (Emms and Kelly, 2019) (https://github.com/davidemms/OrthoFinder) to obtain multiple sequence alignment files. Phylogenetic trees were constructed using IQtree v2.3.6 (Minh et al., 2020)(https://github.com/iqtree/iqtree2) with automatic model selection and 1000 bootstrap replicates. All trees were visualized and annotated using Tree Visualization By One Table (tvBOT) (Xie et al., 2023).

### 2.7 Animal infection experiment

Using a random number method, 6–7 weeks aged female C57BL/6J mice weighing 20–25 g were randomly divided into infection and control groups. Five mice in each infection group were intranasally inoculated with 25 μL of 1.5 × 10^8^ CFU/mL of tested strains, and mice in the control groups were inoculated with PBS. At 4 h, 12 h, 1 day, 2 days and 3 days post-infection, the mice were sacrificed and the lung and spleen tissues were collected aseptically. The established Chinese reference strain of *S. maltophilia* strain 11066 was used as infection control in this experiment (Yue et al., 2023).

The left lobes of the lungs were fixed in 10% neutral formalin for 24 h, followed by tissue processing, paraffin embedding, sectioning and staining, and blindly examined by an expert in the field of laboratory animal pathology. Pulmonary clearance and occurrence of disseminated infection were monitored via quantitative bacteriology of the lung and spleen homogenates, respectively. Briefly, tissues were homogenised (1000 rpm) on ice in 1 mL of sterile normal saline by using a homogeniser (RZ-GR96A; Beijing Guoke Rongzhi Biotechnology Co., Ltd., China), 100 μL 10-fold serial dilutions of homogenates were spread on LB agar. The number of colonies was counted 24–48 h after incubation at 37°C. Bacterial colony counts were normalised according to the wet tissue weight and calculated as CFU/g.

### 2.8 Quantified cytokines tests

Remaining lung homogenates were used for quantified cytokines tests. Nine out of ten cytokines, namely tumour necrosis factor alpha (TNF-α), granulocyte-macrophage colony-stimulating factor (GM-CSF), gamma interferon (IFN-γ), interleukin (IL)-2, IL-4, IL-6, IL-10, IL-12p70, and IL-17A were quantified using Luminex technology (R&D Systems, Minneapolis, MN, USA) and reagents (12002798; BIO-RAD, USA). IL-22 was tested using RayBio^®^ Mouse IL-22 ELISA Kit.

Data are expressed as mean ± standard deviation. The *t*-test is used to compare the differences between the two groups. For the comparison between groups, the analysis of variance with Dunnett’s t-test is used. *P*<0.05 indicates that the difference is statistically significant.

## 3 Results and discussion

### 3.1 Systematic phenotypic characteristics of the strain

The strain 704A1^T^ was isolated from sputum samples of a 34-year-old female. The woman was clinically diagnosed as tuberculous pleurisy and under anti-TB treatment. The γ-interferon release assay test of this patient was positive, but the isolation of *Mycobacterium tuberculosis* from the sputum sample was negative.

**Figure 1:**
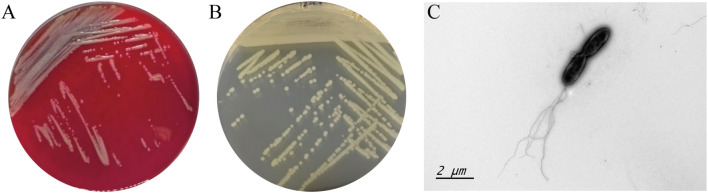
Growth morphology 704A1^T^ on columbia blood agar **(A)**, MH medium **(B)** and the bacterial image under the transmission electron microscopy **(C)**.

704A1^T^ is Gram- negative bacterium and grows well on tryptone soy (TSA; Difco), Luria-Bertani (LB; Difco), columbia blood agar (OXOID) and Mueller-Hinton (MH; Difco). Colorless, transparent, smooth and moist colonies on nutrient agar and grayish white, non-hemolytic colonies with ammonia odor on blood agar can be observed after 18–24 hours (**Figures 1A, B**). Growth of 704A1^T^ on LB medium could be observed under conditions of 15-37 °C, pH range 5-10, and NaCl concentration of 0-5%. Under microscopes, 704A1^T^ cells are Gram negative rod-shaped, 1.5-2 × 0.4-0.6 μM bodies with 1–5 flagella at the single extreme (**Figure 1C**).

Among a total of 84 reactions, 704A1^T^ showed positive results for nitrate reduction, hydrolysis of heptavidin and gelatin, assimilation of β-galactosidase, glucose, mannose, *N-*acetylglucosamine, maltose, malic acid, and citric acid in API 20NE; it was positive for reactions of alkaline phosphatase, esterase lipase (C8), leucine arylamidase, acid phosphatase, and naphthol-AS-BI phosphate hydrolase in API ZYM. The only substrate that 704A1^T^ could use as carbon source was esculin in API 50CH.

704A1^T^ contains all 3 characteristic fatty acids of the genus *Stenotrophomonas*, iso-C_11:0_, iso-C_11:03_ 3OH, and iso-C_13:0_ 3OH (Heylen et al., 2007). Its predominant fatty acids were iso-C_15:0,_ C_15:0_ anteiso, summed Feature 3(C_16:1_ω7c/16:1ω6c) and C_16:0_. The main peaks of 704A1^T^ in the MALDI-TOF spectrum located at 4857.381Da, 5253.734Da, 6071.902Da and 4844.272Da. Both in terms of fatty acid and proteins composition, 704A1^T^ has a high degree of similarity with *S. maltophilia*. The similarity index to *S. maltophilia* in the RTSBA6 library is 0.643, and the identification score in the Bruker_SSP library is 1.985.

### 3.2 Genetic characteristics and species classification

The complete genome of 704A1^T^ was 4,686,432 bp in length and 63.77% GC content. The subreads N50 was 11,437 bp and N90 was 7,180 bp. Genome depth was 45.49%. The statistics of non-coding RNA were 73 tRNAs, 5 5S rRNAs, 4 16S rRNAs, 4 23S rRNAs and 22 sRNAs. A total of 4,214 CDSs, 315 tandem repeats, 214 minisatellite DNAs, and 16 microsatellite DNAs were predicted. Gene function annotation based on eleven databases showed that 704A1^T^ has total 4128 functional genes, including 314 virulence factor genes in VFDB, 179 carbohydrate-active enzymes in CAZY, 3150 proteins in COG, 2548 genes in KEGG and 548 genes in T3SS. Scanning against COG, KEGG, NR, SWISSPROT and VFDB databases indicated that 704A1^T^ has a membrane channel-forming protein YqfA of hemolysin III family on its genome, but no active haemolysin encoding gene like *hlyB* and *hlyIII* of 610A2^T^, the type strain of *Stenotrophomonas pigmentate* which was isolated from tuberculosis patients during the same period of 704A1^T^. The genotype of 704A1^T^ was consistent with its phenotype that the 704A1^T^ did not form hemolytic rings like 610A2^T^ on the blood agar (Li et al., 2024).

**Figure 2:**
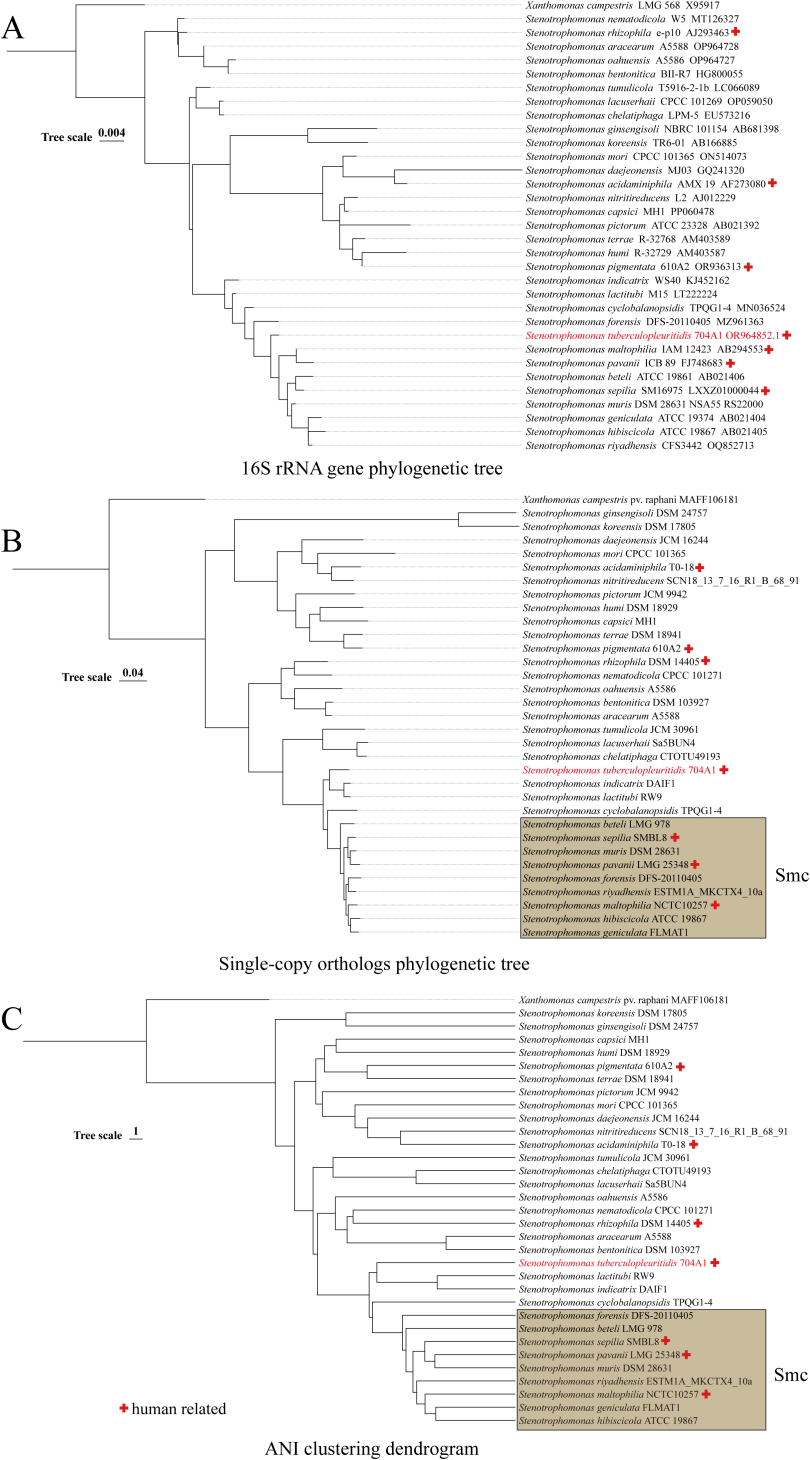
Phylogenetic/clustering tree constructed based on 16S rRNA gene **(A)**, single-copy orthologs **(B)**, and ANI **(C)**.

16S rRNA gene sequencing is the most commonly used method for the preliminary identification of bacterial species. Although its resolution is insufficient to differentiate closely related *Stenotrophomonas* species (Yu and Wang, 2024), it still holds value in indicating potential novel taxa when sequence homology to known genomes is low. On the phylogenetic tree of 16S rRNA gene, 704A1^T^was incorporated into a clade containing 12 species, including *S. maltophilia*, *S. indicatrix*, etc (**Figure 2**). ANI serves as the gold standard for defining prokaryotic species at the genomic level, with established threshold values validated for *Stenotrophomonas* species and complexes (Yu and Wang, 2024). The pairwise ANI comparisons between 704A1^T^ and the 33 *Stenotrophomonas* species generated 18 valid ANI values, ranging from 89.66 and 83.77. with the highest value observed for *S. indicatrix*. Therefore, based on the 95% ANI threshold value suggested for *Stenotrophomonas* (Yu and Wang, 2024), 704A1^T^ can be judged as new species of genus *Stenotrophomonas*. A total of 1,162 core genes were identified from the 33 genomes. On both the ANI clustering tree and single-copy orthologs phylogenetic tree, 704A1^T^ formed a clade adjacent to the *Stenotrophomonas maltophilia* complex (Smc) together with *S. indicatrix* and *S. lactitubi* (**Figure 2**).

### 3.3 Multi-antibiotic resistance

The AST results of the 39 drugs tested showed that 704A1^T^ possessed the common characteristic of the genus *Stenotrophomonas* and is also a multidrug-resistant strain (**Table 1**). 704A1^T^ is sensitivity to tetracyclines, aminoglycosides, and other antibiotics such as chloramphenicol, fosfomycin and polymyxin, but is generally resistant to β-lactam and quinolone antibiotics, while β-lactam inhibitors sulbactam and tazobactam can better increase the sensitivity of 704A1^T^ to β-lactams than clavulanate. It is worth noting that 704A1 ^T^ is resistant to more than half of the anti-TB drugs, including rifampicin and rifabutin. Therefore, while the AST results for *M. tuberculosis* mixed with 704A1^T^ will not be misjudged as MDR-TB like 610A2 ^T^, it will just incorrectly be deemed as rifampicin-resistant (RR)-TB. The effect of its multidrug resistance on AST and treatment in cases of mixed infections stands out as a particularly notable aspect of *Stenotrophomonas*.

**Table 1:** AST results of 704A1^T^.

Classification of antibiotics		Susceptible	Intermediate	Resistant
β-lactams	Cephalosporins	Ceftazidime	Cefepime Cefoxitin	Cefazolin Cefuroxim Ceftriaxone
	Carbapenems			Ertapenem Imipenem Meropenem
	monocyclic β-lactam			Aztreonam
β-lactam/β-lactamase inhibitor combinations		Ampicillin/sulbactam Cefoperazone/sulbactam Piperacillin/tazobactam		Amoxicillin/clavulanate
Tetracyclines		Minocycline Tigecycline	Tetracycline	
Nitrofuran				Nitrofurantoin
Aminoglycosides		Tobramycin Gentamicin Amikacin		kanamycin
Quinolones		Levofloxacin		Norfloxacin Ofloxacin Moxifloxacin Ciprofloxacin
Sulfonamides		Trimethoprim/sulfamethoxazole		
Other		Chloramphenicol Fosfomycin Polymyxin		
Anti-TB drugs		Isoniazid Ethambutol Ethionamide *p*-aminosalicylic acid		Rifampicin Streptomycin Rifabutin Cycloserine

On the 704A1^T^ genome, there are 85 antibiotic resistance genes matched in ARDB, CARD and KEGG, including 13 genes related to the resistance of the beta-lactam antibiotic (*ampC*, *blaI*, *bl3_l*, and *bl2e_y56*, et al), *ksga* gene related to kasugamycin, *qnrb* gene related to fluoroquinolone, *aph6ic* and *aph3va* related to aminoglycosides, *dfra26* related to trimethoprim, and 22 multidrug resistance efflux pump genes. The drug resistance of 704A1^T^ had good phenotype-genotype correlation.

### 3.4 The virulence in mouse infection model

**Figure 3:**
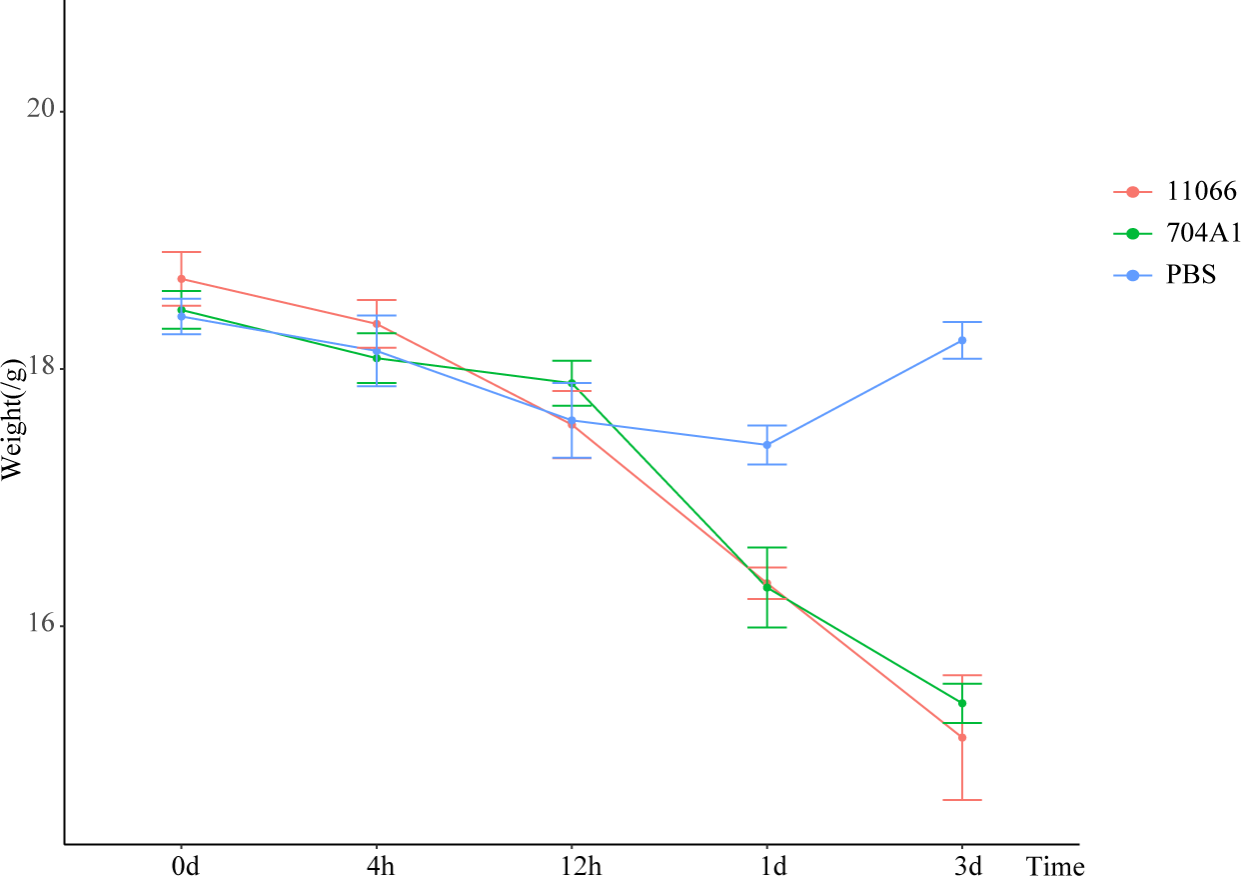
The weight losing curves in mouse infection model.

**Figure 4:**
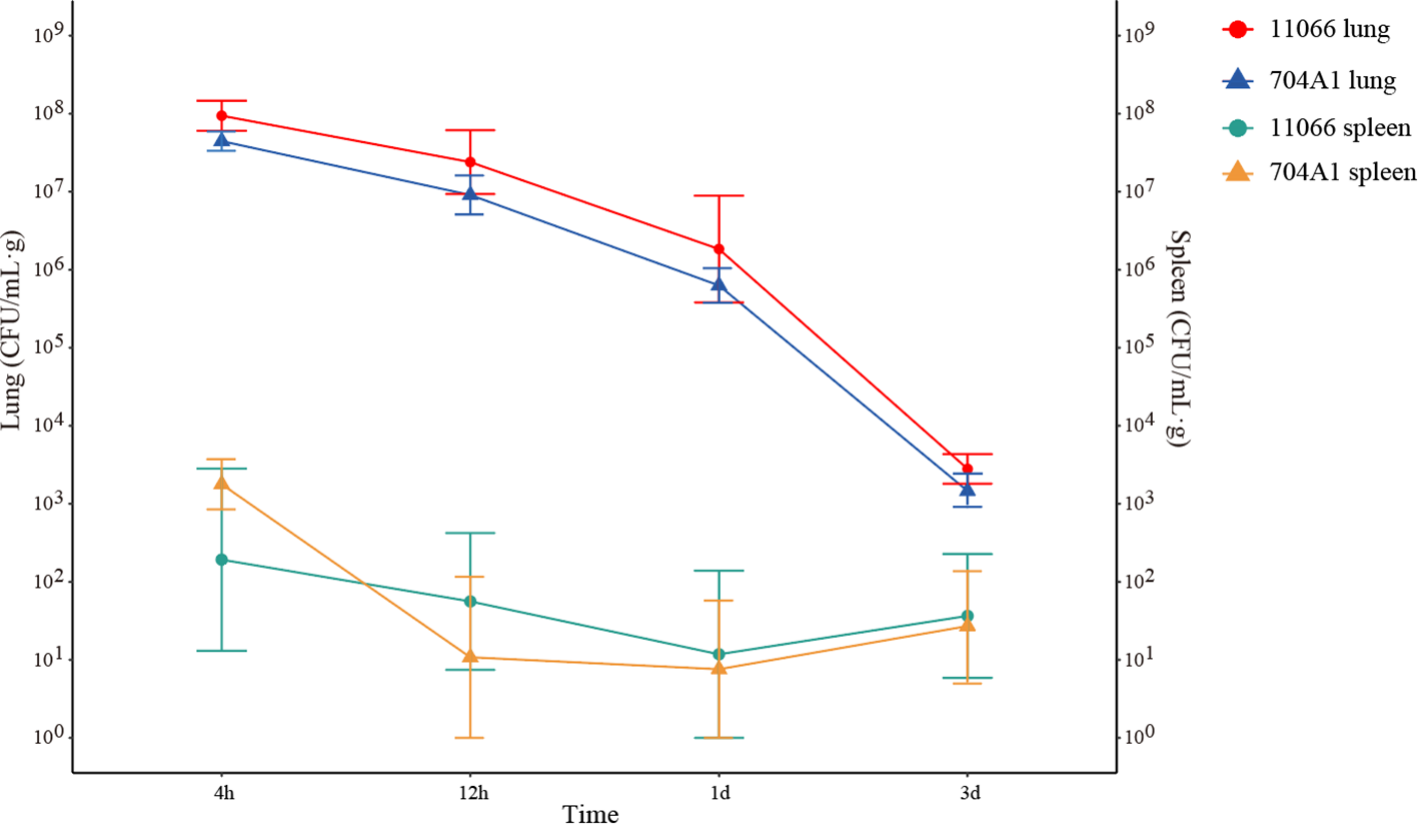
The bacterial load in murine lungs and spleens after infection.

**Figure 5:**
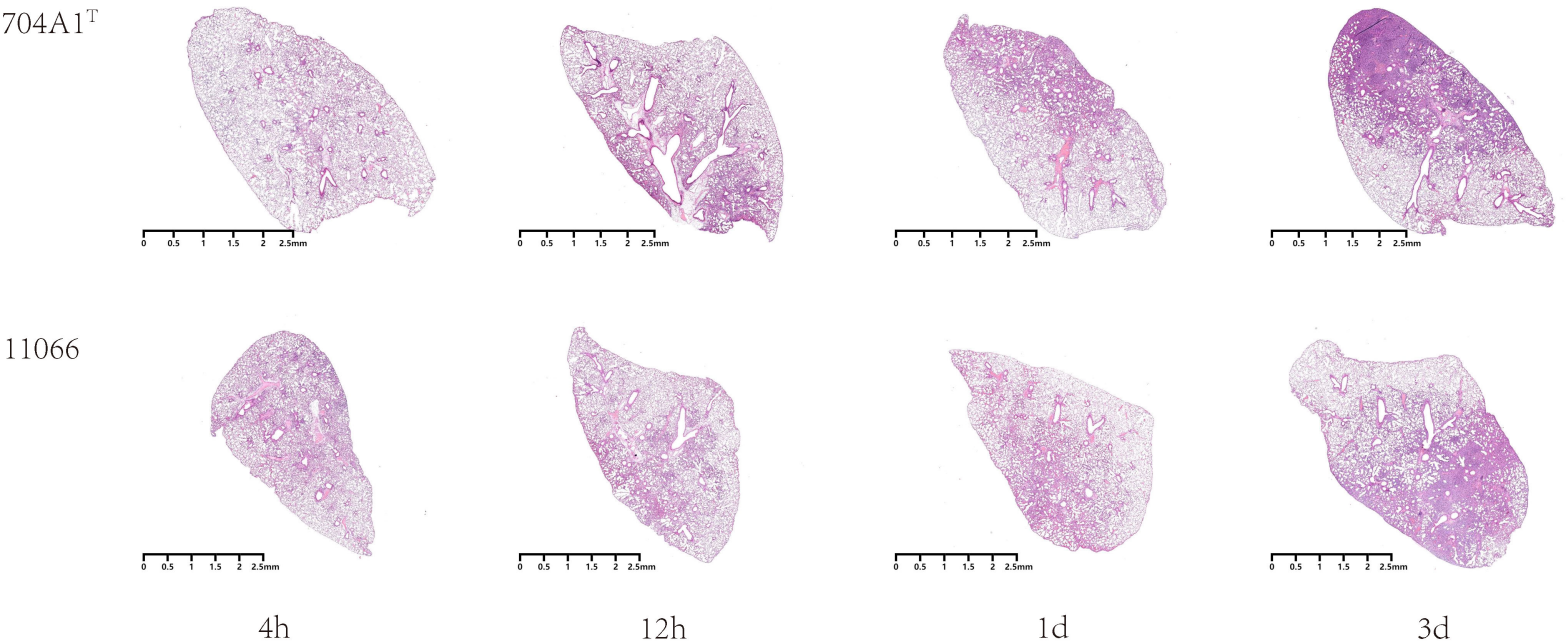
Microscopic images of lung histopathology after infection.

At the intranasal infection dose of 25 μL 1.5 × 10^8^ CFU/mL, no mortality was observed. The body weights of the mice infected with 704A1^T^ manifested a continuous downward trend. By the third day post-infection, the percentage of average weight loss had reached 16.5% (**Figure 3**). The bacterial load of 704A1^T^ per tissue weight (g) in lung at four post-infection time points of 4h, 12h, 1d, and 3d infected were 4.84 ± 2.55×10^8^ CFU/mL·g, 1.18 ± 0.85×10^7^ CFU/mL·g, 7.76 ± 4.96×10^5^ CFU/mL·g, and 1.80 ± 1.21×10^3^ CFU/mL·g respectively (**Figure 4**). The strain could be isolated from the spleen homogenates at all four time points, indicating that 704A1^T^ can invasive and spread in blood. Histological examination showed that pulmonary damage could be obviously observed starting from 12 hours after 704A1^T^ infection. At the 3d post-infection, more than 50% of the lung tissue underwent consolidation (**Figure 5**).

Compared with the infection of *S. maltophilia* strain 11066, there were no statistically significant differences in the changes of body weight, the bacterial loads in the lung and spleen of mice. The changes in histopathology were also quite similar. However, the mice in the group infected with 704A1^T^ exhibited more severe clinical symptoms, including obviously decreased activity, listlessness, rapid shallow breathing and piloerection than those infected with 11066. The infections between 704A1^T^ and 610A2^T^ showed more significant differences than 11066. 610A2^T^ could be cleared from the murine lungs two days after infection, and it could only be isolated from the spleen within four hours. The histopathological findings showed pulmonary hemorrhage without consolidation. Therefore, 704A1^T^ persisted in the host for a longer time and caused more severe damage to the lung tissue than 610A2^T^.

On the 704A1^T^ genome, 314 virulence factors were identified from VFDB, in which 26 factors were classified in level1 human diseases and Level2 infectious disease. Besides 548 genes identified in T3SS, annotation results showed that 704A1^T^ possessed two types of secretion systems, consisting of 5 type IV secretion system genes (*virB1*- *virB4* and *virB11*) and 7 type II secretion system HxcT pseudopilin genes (*hxc Q*, *R*, *S*, *T*, *U*, *V*, *W*). In addition to multiple chemotaxis factors, flagellar and pili related genes, biofilm-controlling response genes, it is worth noting that the 704A1^T^ genome harbors a variety of virulence-related genes. For example, 704A1^T^ harbors two *mip* genes, which encode the macrophage infectivity potentiator. This surface protein with peptidyl‐prolyl‐cis/trans isomerase activity is a kind of conserved virulence factor and plays a critical role in the effective infection of host cells. Its capacity to promote the parasitism of *Legionella pneumophila* within human macrophages and induce pneumonia in experimental models has been reported (Cianciotto et al., 1990; McChlery et al., 2009). The regulator of virulence determinants PhoP, belonging to the two-component system PhoPR, directly regulates the expression of genes such as *moxR*, which associated with the pathogenicity and bacterial load in various tissues of *Riemerella anatipestifer*, the causing bacterium of duck infectious serositis (Zhang et al., 2025). The transcriptional regulator RcsB on the 704A1^T^genome is a regulator that, like PhoP, is regulated by acetylation and involved in various processes such as biofilm formation and cell division (Qin et al., 2016; Lammers, 2021). Moreover, five Dot/Icm type IV secretion system effectors encoding genes were identified on the 704A1^T^genome. This is a system that can translocate more than 300 virulence factors (effectors) into host cells to facility intracellular survival and govern host cell interactions (Schroeder et al., 2010; Weber et al., 2014). 704A1^T^ carries three thioredoxin 1 genes. Trx1 acts as reductases in redox regulation and protects proteins from oxidative aggregation and inactivation. Trx1 helps the cells to cope with various environmental stresses and inhibits programmed cell death. It also plays important roles in suppressing neurodegenerative disorders and resistance against oxidative stress-associated neuron damage (Awan et al., 2022). Some of these virulence genes are harbored by 704A, such as *mip*, but absent in 610A2^T^ and 11066. It is speculated that this may be the reason that the pathogenicity of 704A showed in the mouse infection experiment are stronger than those of 610A2^T^ and 11066, and its longer residence time in mice compared to the latter two.

### 3.5 The immune characteristics

**Figure 6:**
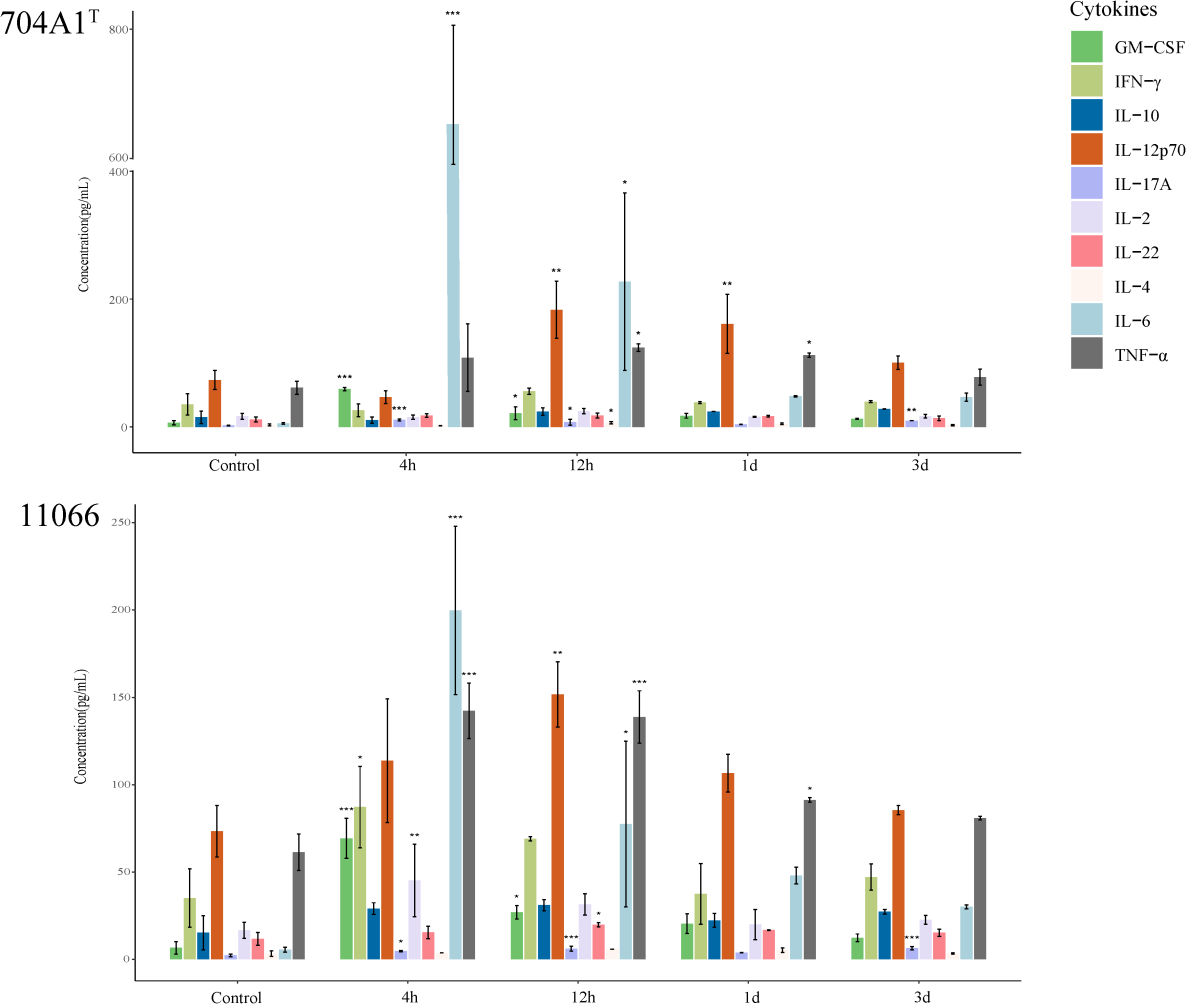
Immune response of 10 cytokines in the lung homogenates after infection of 704A1^T^ and 11066. *P<0.05, **P<0.01, ***P<0.001.

After infection with 704A1, the overall immune response in mice was rapid (**Figure 6**). At 4 hours, among the 10 cytokines detected, GM-CSF, IL-17A, and IL-6 were significantly higher compared to the control group, suggesting that the host was in the early stage of the acute inflammatory response. In particular, the level of IL-6 was as high as 650.75 pg/mL, significantly higher than those in the infections with 11066 and 610A2^T^ (which were 199.83 pg/mL and 115.78 pg/mL respectively). IL-6 has been reported to be related to the severity of many diseases (Schulte et al., 2013). The IL-6 levels in 704A1^T^ are consistent with the fact that among infections by three *Stenotrophomonas* strains, it exhibited the most severe signs and pathological changes, even more severe than 11066, the representative clinical pathogenic species *S. maltophilia*. This result also suggested that IL-6 could serve as an early predictive indicator of the severity of the disease. At 12 hours, the levels of IL-6 and GM-CSF were significantly lower than those at 4 hours, and IL-12p70 began to increase significantly. IL-12p70 is a cytokine that promotes the differentiation of T cells into Th1 type, indicating that the immune system had shifted from the initial non-specific response to a more targeted adaptive immune response and the establishment of the Th1 immune response. One day after infection, the levels of most cytokines returned to normal, and only IL-12p70 and TNF-α were higher than those in the control group, indicating that the acute inflammation had been controlled. Three days after infection, only IL-17A among the cytokines was still higher than that in the control group. IL-17A is mainly produced by Th17 cells and plays an important role in tissue damage repair, which indicates that the acute inflammation has been controlled, and there is a transition to adaptive immunity and tissue damage repair. It is worth mentioning that the level of the cytokine IL-22, which is related to the mucosal barrier function, showed no significant change throughout the whole infection process (Dudakov et al., 2015). This may be because *Stenotrophomonas* strains can quickly invade the body and even spread through the blood, and it is not an infection mainly dominated by mucosal immunity.

Description of *Stenotrophomonas tuberculopleuritidis* sp. nov.

*Stenotrophomonas tuberculopleuritidis* sp. nov. (tu.ber.cu.lo.pleu.ri’ti.dis. N.L. fem. n. *tuberculopleuritis*, tuberculous pleurisy; N.L. gen. n. *tuberculopleuritidis*, of a tuberculopleurisy patient).

The type strain 704A1^T^ (=GDMCC 1.4133=JCM 36489) was first isolated from a tuberculous pleurisy patient. It is Gram-negative bacillus with 1–5 flagella at the single extreme, which can grow under conditions of 15-37 °C, pH range 5-10, and NaCl concentration of 0-5%. 704A1^T^ showed positive results for nitrate reduction, hydrolysis of heptavidin and gelatin, assimilation of β-galactosidase, glucose, mannose, *N-*acetylglucosamine, maltose, malic acid, citric acid, alkaline phosphatase, esterase lipase (C8), leucine arylamidase, acid phosphatase, and naphthol-AS-BI phosphate hydrolase. Its predominant fatty acids were iso-C_15:0,_ C_15:0_ anteiso, summed Feature 3(C_16:1_ω7c/16:1ω6c) and C_16:0_ and the main peaks of 704A1^T^ in the MALDI-TOF spectrum located at 4857.381Da, 5253.734Da, 6071.902Da and 4844.272Da.

The ANI value of 704A1^T^ compared to the defined species of genus *Stenotrophomonas* ranged between 80.03 and 89.6, with the highest for *S. indicatrix* and *S. geniculata*, and the lowest for *S. ginsengisoli.* The complete genome and 16S rRNA gene sequence of 704A1^T^ have been deposited in GenBank under accession numbers CP130831.1 and OR964852, respectively.

## Data Availability

The datasets presented in this study can be found in online repositories. The names of the repository/repositories and accession number(s) can be found below: https://www.ncbi.nlm.nih.gov/genbank/, CP130831.1 https://www.ncbi.nlm.nih.gov/genbank/, OR964852.

## References

[B1] Awan M. U. N. Yan F. Mahmood F. Bai L. Liu J. Bai J. (2022). The functions of thioredoxin 1 in neurodegeneration. Antioxid. Redox Signal 36, 1023–1036. doi: 10.1089/ars.2021.0186, PMID: 34465198

[B2] Brooke J. S. (2012). *Stenotrophomonas maltophilia*: an emerging global opportunistic pathogen. Clin. Microbiol. Rev. 25, 2–41. doi: 10.1128/CMR.00019-11, PMID: 22232370 PMC3255966

[B3] Brooke J. S. Di Bonaventura G. Berg G. Martinez J. L. (2017). Editorial: a multidisciplinary look at *Stenotrophomonas maltophilia*: an emerging multi-drug-resistant global opportunistic pathogen. Front. Microbiol. 8, 1511. doi: 10.3389/fmicb.2017.01511, PMID: 28912755 PMC5583227

[B4] Chao A. Chao A. S. Lin C. Y. Weng C. H. Wu R. C. Yeh Y. M.. (2022). Analysis of endometrial lavage microbiota reveals an increased relative abundance of the plastic-degrading bacteria *Bacillus pseudofirmus* and *Stenotrophomonas rhizophila* in women with endometrial cancer/endometrial hyperplasia. Front. Cell Infect. Microbiol. 12, 1031967. doi: 10.3389/fcimb.2022.1031967, PMID: 36439209 PMC9682088

[B5] Chen J. Zhu Y. Yin M. Xu Y. Liang X. Huang Y. P. (2019). Characterization of maltocin S16, a phage tail-like bacteriocin with antibacterial activity against *Stenotrophomonas maltophilia* and Escherichia coli. J. Appl. Microbiol. 127, 78–87. doi: 10.1111/jam.14294, PMID: 31021024

[B6] Cianciotto N. P. Bangsborg J. M. Eisenstein B. I. Engleberg N. C. (1990). Identification of mip-like genes in the genus Legionella. Infect. Immun. 58, 2912–2918. doi: 10.1128/iai.58.9.2912-2918.1990, PMID: 2387627 PMC313586

[B7] CLSI (2018). Performance standards for susceptibility tesing of *Mycobacteria*, *Nocardia* spp., and other aerobic actinomycetes, M64 (Wayne: The Clinical and Laboratory Standard Institute). 31339680

[B8] CLSI (2023). Performance standards for antimicrobial susceptibility testing, CLSI M100, 33nd Edition (Wayne: The Clinical and Laboratory Standard Institute).

[B9] Dudakov J. A. Hanash A. M. van den Brink M. R. (2015). Interleukin-22: immunobiology and pathology. Annu. Rev. Immunol. 33, 747–785. doi: 10.1146/annurev-immunol-032414-112123, PMID: 25706098 PMC4407497

[B10] Edgar R. C. (2022). Muscle5: High-accuracy alignment ensembles enable unbiased assessments of sequence homology and phylogeny. Nat. Commun. 13, 6968. doi: 10.1038/s41467-022-34630-w, PMID: 36379955 PMC9664440

[B11] Elhalag K. M. Messiha N. A. Emara H. M. Abdallah S. A. (2016). Evaluation of antibacterial activity of *Stenotrophomonas maltophilia* against *Ralstonia solanacearum* under different application conditions. J. Appl. Microbiol. 120, 1629–1645. doi: 10.1111/jam.13097, PMID: 26876282

[B12] Emms D. M. Kelly S. (2019). OrthoFinder: phylogenetic orthology inference for comparative genomics. Genome Biol. 20, 238. doi: 10.1186/s13059-019-1832-y, PMID: 31727128 PMC6857279

[B13] Gao S. Seo J. S. Wang J. Keum Y. S. Li J. Li Q. X. (2013). Multiple degradation pathways of phenanthrene by *Stenotrophomonas maltophilia* C6. Int. Biodeterior. Biodegrad. 79, 98–104. doi: 10.1016/j.ibiod.2013.01.012, PMID: 23539472 PMC3607548

[B14] Gautam V. Patil P. P. Bansal K. Kumar S. Kaur A. Singh A.. (2021). Description of *Stenotrophomonas sepilia* sp. nov., isolated from blood culture of a hospitalized patient as a new member of *Stenotrophomonas maltophilia* complex. New Microbes New Infect. 43, 100920. doi: 10.1016/j.nmni.2021.100920, PMID: 34457314 PMC8379335

[B15] Gil-Gil T. Martinez J. L. Blanco P. (2020). Mechanisms of antimicrobial resistance in *Stenotrophomonas maltophilia*: a review of current knowledge. Expert Rev. Anti Infect. Ther. 18, 335–347. doi: 10.1080/14787210.2020.1730178, PMID: 32052662

[B16] Heylen K. Vanparys B. Peirsegaele F. Lebbe L. De Vos P. (2007). *Stenotrophomonas terrae* sp. nov. and *Stenotrophomonas humi* sp. nov., two nitrate-reducing bacteria isolated from soil. Int. J. Syst. Evol. Microbiol. 57, 2056–2061. doi: 10.1099/ijs.0.65044-0, PMID: 17766871

[B17] Huang X. Liu J. Ding J. He Q. Xiong R. Zhang K. (2009). The investigation of nematocidal activity in *Stenotrophomonas maltophilia* G2 and characterization of a novel virulence serine protease. Can. J. Microbiol. 55, 934–942. doi: 10.1139/W09-045, PMID: 19898533

[B18] Iebba V. Guerrieri F. Di Gregorio V. Levrero M. Gagliardi A. Santangelo F.. (2018). Combining amplicon sequencing and metabolomics in cirrhotic patients highlights distinctive microbiota features involved in bacterial translocation, systemic inflammation and hepatic encephalopathy. Sci. Rep. 8, 8210. doi: 10.1038/s41598-018-26509-y, PMID: 29844325 PMC5974022

[B19] Jain C. Rodriguez R. L. Phillippy A. M. Konstantinidis K. T. Aluru S. (2018). High throughput ANI analysis of 90K prokaryotic genomes reveals clear species boundaries. Nat. Commun. 9, 5114. doi: 10.1038/s41467-018-07641-9, PMID: 30504855 PMC6269478

[B20] Jakobi M. Winkelmann G. Kaiser D. Kempler C. Jung G. Berg G.. (1996). Maltophilin: a new antifungal compound produced by *Stenotrophomonas maltophilia* R3089. J. Antibiot. 49, 1101–1104. doi: 10.7164/antibiotics.49.1101, PMID: 8982338

[B21] Jezek P. Safrankova R. Malisova L. (2020). Unusual microbiological findings in bacteremia cases - three case reports. Klin Mikrobiol. Infekc. Lek. 26, 106–110., PMID: 33418599

[B22] Kumar S. Bansal K. Patil P. P. Kaur A. Kaur S. Jaswal V.. (2020). Genomic insights into evolution of extensive drug resistance in *Stenotrophomonas maltophilia* complex. Genomics 112, 4171–4178. doi: 10.1016/j.ygeno.2020.06.049, PMID: 32653516

[B23] Kumar A. Rithesh L. Kumar V. Raghuvanshi N. Chaudhary K. Abhineet. (2023). *Stenotrophomonas* in diversified cropping systems: friend or foe? Front. Microbiol. 14, 1214680. doi: 10.3389/fmicb.2023.1214680, PMID: 37601357 PMC10437078

[B24] Lammers M. (2021). Post-translational lysine ac(et)ylation in bacteria: a biochemical, structural, and synthetic biological perspective. Front. Microbiol. 12, 757179. doi: 10.3389/fmicb.2021.757179, PMID: 34721364 PMC8556138

[B25] Li Y. Yu Z. Fan X. Xu D. Liu H. Zhao X.. (2024). A novel pathogenic species of genus *Stenotrophomonas: Stenotrophomonas pigmentata* sp. nov. Front. Cell Infect. Microbiol. 14, 1410385. doi: 10.3389/fcimb.2024.1410385, PMID: 38903940 PMC11188353

[B26] Li Y. Zhao A. Yu Q. Yu N. Cui Y. Ma X.. (2023). Effect of *Stenotrophomonas maltophilia* on tuberculosis. Microbiol. Spectr. 11, e0094423. doi: 10.1128/spectrum.00944-23, PMID: 37306591 PMC10433947

[B27] Ling S. Zhao Y. Sun S. Zheng D. Sun X. Zeng R.. (2022). Enhanced anti-herbivore defense of tomato plants against *Spodoptera litura* by their rhizosphere bacteria. BMC Plant Biol. 22, 254. doi: 10.1186/s12870-022-03644-3, PMID: 35606741 PMC9128215

[B28] Liu J. Chen P. Zheng C. Huang Y. P. (2013). Characterization of maltocin P28, a novel phage tail-like bacteriocin from Stenotrophomonas maltophilia. Appl. Environ. Microbiol. 79, 5593–5600. doi: 10.1128/AEM.01648-13, PMID: 23835182 PMC3754179

[B29] Macori G. Al-Qahtani A. A. Koolman L. Althawadi S. Mutabaqani M. Bashtawi R.. (2024). *Stenotrophomonas riyadhensis* sp. nov., isolated from a hospital floor swab. Int. J. Syst. Evol. Microbiol. 74. doi: 10.1099/ijsem.0.006250, PMID: 38393318

[B30] McChlery S. Ramage G. Bagg J. (2009). Respiratory tract infections and pneumonia. Periodontol. 2000 49, 151–165. doi: 10.1111/j.1600-0757.2008.00278.x, PMID: 19152532 PMC7168030

[B31] Menetrey Q. Sorlin P. Jumas-Bilak E. Chiron R. Dupont C. Marchandin H. (2021). *Achromobacter xylosoxidans* and *Stenotrophomonas maltophilia*: emerging pathogens well-armed for life in the cystic fibrosis patients’ lung. Genes (Basel) 12(5):610–31. doi: 10.3390/genes12050610, PMID: 33919046 PMC8142972

[B32] Minh B. Q. Schmidt H. A. Chernomor O. Schrempf D. Woodhams M. D. von Haeseler A.. (2020). IQ-TREE 2: new models and efficient methods for phylogenetic inference in the genomic era. Mol. Biol. Evol. 37, 1530–1534. doi: 10.1093/molbev/msaa015, PMID: 32011700 PMC7182206

[B33] Nakayama T. Homma Y. Hashidoko Y. Mizutani J. Tahara S. (1999). Possible role of xanthobaccins produced by *Stenotrophomonas* sp. strain SB-K88 in suppression of sugar beet damping-off disease. Appl. Environ. Microbiol. 65, 4334–4339. doi: 10.1128/AEM.65.10.4334-4339.1999, PMID: 10508056 PMC91574

[B34] Palleroni N. J. Bradbury J. F. (1993). *Stenotrophomonas*, a new bacterial genus for *Xanthomonas maltophilia* (Hugh 1980) Swings et al., 1983. Int. J. Syst. Bacteriol. 43, 606–609. doi: 10.1099/00207713-43-3-606, PMID: 8347518

[B35] Paškevičius Š. Gleba Y. Ražanskienė A. (2022). Stenocins: novel modular bacteriocins from opportunistic pathogen *Stenotrophomonas maltophilia* . J. Biotechnol. 351, 9–12. doi: 10.1016/j.jbiotec.2022.04.006, PMID: 35436577

[B36] Qin R. Sang Y. Ren J. Zhang Q. Li S. Cui Z.. (2016). The bacterial two-hybrid system uncovers the involvement of acetylation in regulating of lrp activity in Salmonella Typhimurium. Front. Microbiol. 7, 1864. doi: 10.3389/fmicb.2016.01864, PMID: 27909434 PMC5112231

[B37] Sanchez M. B. (2015). Antibiotic resistance in the opportunistic pathogen Stenotrophomonas maltophilia. Front. Microbiol. 6, 658. doi: 10.3389/fmicb.2015.00658, PMID: 26175724 PMC4485184

[B38] Schroeder G. N. Petty N. K. Mousnier A. Harding C. R. Vogrin A. J. Wee B.. (2010). *Legionella pneumophila* strain 130b possesses a unique combination of type IV secretion systems and novel Dot/Icm secretion system effector proteins. J. Bacteriol. 192, 6001–6016. doi: 10.1128/JB.00778-10, PMID: 20833813 PMC2976443

[B39] Schulte W. Bernhagen J. Bucala R. (2013). Cytokines in sepsis: potent immunoregulators and potential therapeutic targets–an updated view. Mediators Inflamm. 2013, 165974. doi: 10.1155/2013/165974, PMID: 23853427 PMC3703895

[B40] Tamma P. D. Aitken S. L. Bonomo R. A. Mathers A. J. van Duin D. Clancy C. J. (2022). Infectious Diseases Society of America Guidance on the treatment of ampC β-lactamase-producing *Enterobacterales*, carbapenem-resistant *Acinetobacter baumannii*, and Stenotrophomonas maltophilia Infections. Clin. Infect. Dis.: an Off. Publ. Infect. Dis. Soc. America 74, 2089–2114. doi: 10.1093/cid/ciab1013, PMID: 34864936

[B41] Tan R. Liu J. Li M. Huang J. Sun J. Qu H. (2014). Epidemiology and antimicrobial resistance among commonly encountered bacteria associated with infections and colonization in intensive care units in a university-affiliated hospital in Shanghai. Wei mian yu gan ran za zhi 47, 87–94. doi: 10.1016/j.jmii.2012.11.006, PMID: 23357606

[B42] Trifonova A. Strateva T. (2019). *Stenotrophomonas maltophilia* - a low-grade pathogen with numerous virulence factors. Infect. Dis. (London England) 51, 168–178. doi: 10.1080/23744235.2018.1531145, PMID: 30422737

[B43] Weber S. Stirnimann C. U. Wieser M. Frey D. Meier R. Engelhardt S.. (2014). A type IV translocated Legionella cysteine phytase counteracts intracellular growth restriction by phytate. J. Biol. Chem. 289, 34175–34188. doi: 10.1074/jbc.M114.592568, PMID: 25339170 PMC4256350

[B44] Xie J. Chen Y. Cai G. Cai R. Hu Z. Wang H. (2023). Tree Visualization By One Table (tvBOT): a web application for visualizing, modifying and annotating phylogenetic trees. Nucleic Acids Res. 51, W587–WW92. doi: 10.1093/nar/gkad359, PMID: 37144476 PMC10320113

[B45] Yu Z. Wang R. (2024). Revised taxonomic classification of the *Stenotrophomonas* genomes, providing new insights into the genus Stenotrophomonas. Front. Microbiol. 15, 1488674. doi: 10.3389/fmicb.2024.1488674, PMID: 39726962 PMC11669713

[B46] Yue L. Yu Z. Wang R. (2023). Establishment and evaluation of a reference strain of Stenotrophomonas maltophilia. Dis. Surveill. 38, 1484–1490.

[B47] Zhang Y. Li D. Yan Q. Xu P. Chen W. Xin H.. (2022). Genome-wide analysis reveals the emergence of multidrug resistant *Stenotrophomonas acidaminiphila* strain SINDOREI isolated from a patient with sepsis. Front. Microbiol. 13, 989259. doi: 10.3389/fmicb.2022.989259, PMID: 36212813 PMC9537462

[B48] Zhang Y. Zhang Y. He Y. Hou Y. Li X. Yang X.. (2025). MoxR effects as an ATPase on anti-stress and pathogenicity of Riemerella anatipestifer. Vet. Res. 56, 44. doi: 10.1186/s13567-025-01454-7, PMID: 39962505 PMC11834572

